# Scanning probe microscopy for energy-related materials

**DOI:** 10.3762/bjnano.10.12

**Published:** 2019-01-10

**Authors:** Rüdiger Berger, Benjamin Grévin, Philippe Leclère, Yi Zhang

**Affiliations:** 1 Max Planck Institute for Polymer Research, Mainz, Germany; 2 UMR5819 CEA-CNRS-UGA, CEA-Grenoble INAC/SYMMES, Grenoble, France; 3 CIRMAP, University of Mons (UMONS), Mons, Belgium; 4 Shanghai Institute of Applied Physics, Chinese Academy of Sciences, Shanghai, PR of China

**Keywords:** energy conversion and storage, in-operando, Scanning Probe Microscopy, Scanning Force Microscopy

In order to stimulate, bundle and strengthen the activities in the field of scanning probe microscopy for energy applications, we have organized a symposium at the European Material Research Society (E-MRS) fall meeting held in Warsaw in 2017. We were impressed by the high quality of the presentations and decided to create this thematic issue published in the Beilstein Journal of Nanotechnology based on these results. We feel that the manuscripts perfectly reflect the current activities and advances in the field of scanning probe microscopy for energy applications*.*

The term “energy applications” refers to materials that are used for energy conversion, energy transport and energy storage. In these fields, intensive basic and applied research is ongoing to address requirements of today and the future. These requirements are, for example, high power conversion efficiency, loss-free transport of energy, fast charging rates and high charging capacity. In order to fulfil these requirements, specific functional materials are being developed, investigated and optimized. Energy-related materials often include electrochemical reactions and (opto-)electronic transport phenomena at their interfaces. In particular, material properties on the nanometer scale play a major role. The understanding of these nanoscale phenomena occurring at material interfaces is therefore essential. Furthermore, these interface phenomena are strongly linked to material properties such as grain size, roughness, mechanical properties and work function. In an attempt to address the diversity of phenomena on the nanoscale, scanning probe microscopy (SPM) methods play an significant role for the in-operando characterization. SPM methods offer a plethora of operation modes beyond topography imaging, which is well reflected in the articles of this thematic issue.

The majority of contributions stem from research on photovoltaic materials. Here, electrical conductive atomic force microscopy (cAFM) and Kelvin probe force microscopy (KPFM) are the major methods that enable the study of the movement of charge carriers and their pathways [1]. We note that the KPFM method is rapidly becoming a tool capable of time-resolved studies. In this context, Yann Almadori and co-workers discuss the time-dependent changes of the surface potential occurring under illumination. This work also unravels lattice expansion phenomena under illumination in perovskite structure forming photo-absorbing materials [2]. Pablo A. Fernández Garrillo and co-workers go one step further by addressing photocarrier dynamics in order to study charge carrier lifetimes. This contribution focuses on a mathematical model to calculate time constants [3]. Such a model is critical for understanding the photophysics at the nanometer scale. Amelie Axt and co-workers discuss the applicability and reliability of different ways of performing KPFM measurements on nanoscale electrical devices [4]. In particular, the knowledge of the true potential of surfaces is required for the analysis of cross-sections of solar cell devices [5–6]. Thus, this work is the basis for future quantitative analysis of nanoscale devices even beyond the scope of solar cells. Katherine Atamanuk and co-workers impressively demonstrate that SPM methods can also be used to perform tomography [7]. They apply photoconducting scanning force microscopy for mapping the open-circuit voltage of cadmium telluride (CdTe) polycrystalline thin film solar cells. Tomography is achieved by gradually removing surface material during continuous high-load topographic imaging. For photovoltaic materials, the interface between materials accepting electrons or holes is of crucial importance. Laurie Letertre and co-workers study a nanocolumnar TiO_2_ surface covalently grafted with a monolayer of poly(3-hexylthiophene) functionalized with carboxylic groups [8]. Their study unravels the physical mechanisms taking place locally during the photovoltaic process and its correlation to the nanoscale morphology.

Electrochemical energy storage (i.e., in a battery) is a major topic in our daily life. Jonathan Op de Beeck and co-workers identify the ionic processes occurring inside Li-ion composites in order to understand the impact on the entire battery cell [9]. In particular, the authors combine cAFM and secondary-ion mass spectrometry to correlate the presence of nanometer-sized conductive paths with the Li concentration. This study exemplifies that SPM combined with complementary methods providing information on the chemistry or atomic composition of materials is very beneficial for understanding the performance of devices. Nino Schön and co-workers study the relationship between Li-ion conductivity and the microstructure of the solid-state electrolyte lithium aluminum titanium phosphate films [10]. Furthermore, dielectric properties play a role for the storage of electrochemical energy. Ying Wang and co-workers report on a novel method for the characterization of the local dielectric distribution based on surface adhesion mapping by SPM [11]. This method is evidently easy in terms of operation and thus has the potential to be widely used. Finally, we want to highlight the contribution “Electrostatic force spectroscopy revealing the degree of reduction of individual graphene oxide sheets” by Yue Shen and co-workers. Yue Shen won the prize for the best presentation during the E-MRS conference [12]. Electrostatic force spectroscopy (EFS) is used here to characterize the degree of reduction of uniformly reduced one-atom-thick graphene oxide (GO) sheets at the nanoscale. The identification and chemical control of the degree of reduction of GO sheets is highly desired to realize nanoscale electronic devices in the future.

We thank all authors for participating with their contribution to this thematic issue. The published manuscripts will be a significant contribution to the advancement of the field of understanding energy materials on the nanometer scale. In addition, we acknowledge the expertise of the reviewers who provided helpful reports to us and the authors. Finally, we acknowledge the valuable support of Wendy Patterson and her team working for the Beilstein Journal of Nanotechnology.

Rüdiger Berger, Benjamin Grévin, Philippe Leclère and Yi Zhang

Mainz, Grenoble, Mons and Shanghai, October 2018
